# Letter to the Editor

**DOI:** 10.3402/gha.v6i0.21787

**Published:** 2013-07-18

**Authors:** Joachim Osur, Traci L. Baird, Brooke A. Levandowski, Emily Jackson, Daniel Murokora

**Affiliations:** 1 Ipas African Alliance, Kenya; 2 Ipas, Chapel Hill, USA; 3 Independent Consultant, Los Angeles, USA; 4 Uganda Women’s Health Initiative, Uganda

We sincerely appreciate Moran and colleagues’ contributions to the discussion about implementing misoprostol for postabortion care (PAC). Many organizations are now working on this issue and sharing information and best practices, as Moran et al. did in their letter, is beneficial to the whole field. They underscore key issues, especially around stakeholder support and sustainable availability of misoprostol. Their data from Zimbabwe about providers’ perception of women's preferences are significant, both because those providers also see the need for women to have options for care, and because it is another example – like ours – of providers being the voices for women. Currently, data related to women's perspectives regarding misoprostol for PAC (MPAC) are limited to acceptability of this service (1–5). While acceptability of MPAC is clearly necessary, understanding women's preferences for PAC treatment modalities is an important area for future research, so services can best meet women's needs. Moran et al. additionally highlight the importance of ensuring that information presented to patients regarding MPAC is not only scientifically accurate but also culturally appropriate.

We support Moran et al.'s point about introducing misoprostol in sites which do not offer manual vacuum aspiration (MVA) and add that a functional referral system must be established for women who may choose vacuum aspiration, who are not eligible for misoprostol, or those who may develop complications that require vacuum aspiration as an intervention. This is consistent with guidance by the World Health Organization (6) but was not part of the design in Kenya where implementation was being done on a pilot basis and before WHO made the recommendation. The model that is promising for the future is to have a referral centre with both services (MVA and MPAC) being fed with referrals from smaller centres that provide MPAC only. Given that misoprostol is inexpensive, stable at room temperature, and easy to use, we expect further expansion of MPAC into sites where it is the sole method available in the future. There is much to be learned by studying such an implementation, particularly across different facilities and among different cadres of health providers.

*Joachim Osur*, MBChB, MPH, PhD Ipas African Alliance, Kenya*Traci L. Baird*, MPH Ipas, Chapel Hill, USA*Brooke A. Levandowski*, PhD, MPA Ipas, Chapel Hill, USA*Emily Jackson*, MD, MPH Independent Consultant Los Angeles, USA*Daniel Murokora*, MB ChB, MMed Uganda Women's Health Initiative Uganda
